# Supplementary material for: Biochemical and functional characterization of a meiosis-specific Pch2/ORC AAA+ assembly
Source: Life Sci Alliance. 2020 Aug 21;3(11):e201900630. doi: 10.26508/lsa.201900630 (PMC7442955; doi:10.26508/lsa.201900630)
Supplement: Supplementary file 4 [file LSA-2019-00630_Supplemental_Data_1.docx]

Supplementary Material

Yeast strains

All strains, except yGV864 and derivatives, are derived from the SK1 background.

yGV48  *MATa, ho::LYS2, lys2, leu2::hisG, his4X::LEU2-URA3, ura3, arg4-nsp, dmc1Δ::ARG4*

MATalpha, ho::LYS2, lys2, leu2::hisG, his4B::LEU2, ura3, arg4-Bgl2, dmc1Δ::ARG4

yGV49 *MATa, ho::LYS2, lys2, ura3, leu2::hisG, his4B::LEU2, arg4-Bgl II*

MATalpha, ho::LYS2, lys2, ura3, leu2::hisG, his4X::LEU2 (Bam)-URA3, arg4-Nsp

yGV864 *MATa, ura3-52, leu2-3, his3, trp1, gal4del, gal80del, GAL2-ADE2, LYS2::GAL1-HIS3, met2::GAL7-lacZ,* yGV933 *MATa, ho::LYS2, lys2, ura3, leu2::hisG, trp1::hisG, his3::hisG*

his4B::LEU2, arg4-Bgl II, pch2::URA3:pPCH2(300bp):3HA-PCH2

MATalpha, ho::LYS2, lys2, ura3, leu2::hisG, trp1::hisG, his3::hisG

his4B::LEU2, arg4-Bgl II, pch2::URA3:pPCH2(300bp):3HA-PCH2

yGV1185 *MATa, ho::LYS2, lys2, ura3, leu2::hisG, TRP1, HIS3,  arg4-Bgl II, pch2::URA3:pPCH2(300bp):3HA-PCH2, orc1::orc1-161 (ts-allele)*

MATalpha, ho::LYS2, lys2, ura3, leu2::hisG, TRP1, ARG, HIS3

his4B::LEU2, pch2::URA3:pPCH2(300bp):3HA-PCH2, orc1::orc1-161

yGV1192 *MATalpha, ho::LYS2, lys2, ura3, leu2::hisG, TRP, his3::hisG,* his4B::LEU2, arg4-Bgl II/nspI

dmc1Δ::ARG4, pch2Δ::KanMX, Cdc6::KanMX6::Pscc1: CDC6

MATa, ho::LYS2, lys2, ura3, leu2::hisG, TRP, arg4-Bgl II/Nsp, his4X::LEU2-(Bam)-URA3, arg4-Nsp, pch2Δ::KanMX4, dmc1Δ::ARG4, Cdc6::KanMX6::Pscc1: CDC6

yGV1269 *MATa, ho::LYS2, lys2, ura3, leu2::hisG, TRP, his4B::LEU2, arg4-Bgl II, dmc1Δ::ARG4, pch2Δ::KanMX4*

MATalpha, ho::LYS2, lys2, ura3, leu2::hisG, TRP, his4B::LEU2, arg4-BglII, dmc1Δ::ARG4, pch2Δ::KanMX4

yGV1506 *MATa, ho::LYS2, lys2, ura3, leu2::hisG, HIS3, pch2::URA3:pPCH2(300bp):3HA-PCH2, orc1::ORC1-TAP::HIS3*

MATalpha, ho::LYS2, lys2, ura3, leu2::hisG, TRP1, HIS3, his4B::LEU2, pch2::URA3:pPCH2(300bp):3HA-PCH2, orc1::ORC1-TAP::HIS3

yGV1508  *MATa, ho::LYS2, lys2, ura3, leu2::hisG, HIS3, trp1::hisG, his4B::LEU2, orc2::ORC2-TAP::HIS3, pch2::URA3:pPCH2(300bp):3HA-PCH2*

MATalpha, ho::LYS2, lys2, ura3, leu2::hisG, HIS3, trp1::hisG, orc2::ORC2-TAP::HIS3, pch2::URA3:pPCH2(300bp):3HA-PCH2

yGV1537  *MATa, ho::LYS2, lys2, ura3, leu2::hisG, TRP1, HIS3, ARG4, ORC5-TAP::HIS3, pch2::URA3:pPCH2(300bp):3HA-PCH2*

MATalpha, ho::LYS2, lys2, ura3, leu2::hisG, trp1::hisG, his3::hisG, ARG4, his4B::LEU2, ORC5-TAP::HIS3, pch2::URA3:pPCH2(300bp):3HA-PCH2

yGV1945 *MATa, ho::LYS2, lys2, ura3, leu2::hisG, HIS3, trp1::hisG, his4B::LEU2, orc2::ORC2-TAP::HIS3, pch2::URA3:pPCH2(300bp):3HA-PCH2, orc1::orc1-161*

*MATalpha, ho::LYS2, lys2, ura3, leu2::hisG, HIS3, trp1::hisG, his4B::LEU2, orc2::ORC2-TAP::HIS3, pch2::URA3:pPCH2(300bp):3HA-PCH2, orc1::orc1-161*

yGV1966  *MATa, ho::LYS2, lys2, ura3, leu2::hisG, ARG4, TRP1, HIS3, orc1::ORC1-TAP::HIS3, pch2::URA3:pPCH2(300bp):3HA-pch2-K320R*

*MATalpha, ho::LYS2, lys2, ura3, leu2::hisG, arg4-bglII, TRP1, HIS3, his4B::LEU2, orc1::ORC1-TAP::HIS3, pch2::URA3:pPCH2(300bp):3HA- pch2-K320R*

yGV2030 *yGV864, [pGAD], [pGBD]*

yGV2036 *yGV864, [pGAD], [pGBD-PCH2]*

yGV2060 *yGV864, [pGAD], [pGBD-PCH2 1-242]*

yGV2061 *yGV864, [pGAD], [pGBD-PCH2-242-565]*

yGV2085 *MATa, ho::LYS2, lys2, ura3, leu2::hisG, TRP,1 HIS3, arg4-Bgl II pch2::URA3:pPCH2(300bp):3HA-PCH2-E399Q****,*** *orc1::ORC1- TAP::HIS3,*

*MATalpha, ho::LYS2, lys2, ura3, leu2::hisG, TRP1, HIS3*

*his4B::LEU2, arg4-Bgl II pch2::URA3:pPCH2(300bp):3HA-PCH2- E399Q****,*** *orc1::ORC1-TAP::HIS3*

yGV2086 *MATalpha, ho::LYS2, lys2, ura3, leu2::hisG, TRP1, HIS3,*

*his4B::LEU2, arg4-Bgl II, pch2::URA3:pPCH2(300bp):3HA-PCH2- E399Q*

*MATa, ho::LYS2, lys2, ura3, leu2::hisG, TRP1, his4B::LEU2, arg4-Bgl II, pch2::URA3:pPCH2(300bp):3HA-PCH2-E399Q*

yGV2114 *yGV864, [pGAD-ORC1], [pGBD]*

yGV2115 *yGV864, [pGAD-ORC1], [pGBD-PCH2-1-242]*

yGV2116 *yGV864, [pGAD-ORC1], [pGBD-PCH2-242-565]*

yGV2117 *yGV864, [pGAD-ORC1], [pGBD-PCH2]*

yGV2155 *MATa, ho::LYS2, lys2, ura3, leu2::hisG, TRP1, HIS3, ARG4,*

*pch2::URA3:pPCH2(300bp):3HA-PCH2-E399Q, orc2::ORC2-TAP::HIS3 MATalpha, ho::LYS2, lys2, ura3, leu2::hisG, TRP1, HIS3, ARG4, his4B::LEU2, pch2::URA3:pPCH2(300bp):3HA-PCH2-E399Q, orc2::ORC2-TAP::HIS3*

yGV2156  *MATa, ho::LYS2, lys2, ura3, leu2::hisG, TRP1, HIS3, arg4-Bgl II,*

*pch2::URA3:pPCH2(300bp):3HA-PCH2-E399Q, ORC5-TAP::HIS3*

*MATalpha, ho::LYS2, lys2, ura3, leu2::hisG, TRP1, HIS3, arg4-Bgl II, pch2::URA3:pPCH2(300bp):3HA-PCH2-E399Q, ORC5-TAP::HIS3*

yGV2203 *MATa, ho::LYS2, lys2, ura3, leu2::hisG, his3::hisG, trp1::hisG,  RPL13A- 2XFKBP12::TRP1, fpr1Δ::KanMX4, tor1- 1::HIS3, ORC2-FRB::KanMX6*

*MATalpha, ho::LYS2, lys2, ura3, leu2::hisG, his3::hisG, trp1::hisG,  RPL13A-2XFKBP12::TRP1, fpr1Δ::KanMX4, tor1- 1::HIS3, ORC2-FRB::KanMX6*

yGV2315 *yGV864, [pGAD-ORC1], [pGBD-PCH2-1-194]*

yGV2316 *yGV864, [pGAD-Orc1], [pGBD-PCH2-1-144]*

yGV2321 *yGV864, [pGAD], [pGBD-PCH2-1-194]*

yGV2322 *yGV864, [pGAD], [pGBD-PCH2-1-144]*

yGV2345  *MATa, ho::LYS2, lys2, leu2::hisG, his4X::LEU2-URA3, ura3, arg4(-nsp),*

*TRP1, dmc1Δ::ARG4, ctf19Δ::KanMX6, cdc6::KanMX6::pSCC1:CDC6*

*MATalpha, ho::LYS2, lys2, leu2::hisG, ura3, arg4(-nsp), dmc1Δ::ARG4, cdc6::KanMX6::pSCC1:CDC6*

yGV2366  *MATa, ho::LYS2, lys2, ura3, leu2::hisG, his3::hisG, arg4, trp1::hisG, RPL13A-2XFKBP12::TRP1, fpr1::KanMX4, tor1-1::HIS3, ORC2- FRB::KanMX6, dmc1Δ::ARG4*

*MATalpha, ho::LYS2, lys2, ura3, leu2::hisG, his3::hisG, arg4, trp1::hisG, RPL13A-2XFKBP12::TRP1, fpr1::KanMX4, tor1-1::HIS3, ORC2- FRB::KanMX6, dmc1Δ::ARG4*

yGV2367  *MATa, ho::LYS2, lys2, leu2::hisG, ura3, arg4-Bgl2, his3::hisG, trp1::hisG, RPL13A-2XFKBP12::TRP1, fpr1::KanMX4, tor1-1::HIS3, dmc1Δ::ARG4*

*MATalpha, ho::LYS2, lys2, leu2::hisG, ura3, arg4-Bgl2, his3::hisG, trp1::hisG, RPL13A-2XFKBP12::TRP1, fpr1::KanMX4, tor1-1::HIS3, dmc1Δ::ARG4*

yGV2393 *MATa, ho::LYS2, lys2, leu2::hisG, ura3, arg4-Bgl2, his3::hisG, trp1::hisG, RPL13A-2XFKBP12::TRP1, fpr1::KanMX4, tor1-1::HIS3, ORC5- FRB::KanMX6, dmc1Δ::ARG4*

*MATalpha, ho::LYS2, lys2, leu2::hisG, ura3, arg4-Bgl2, his3::hisG, trp1::hisG, RPL13A-2XFKBP12::TRP1, fpr1::KanMX4, tor1-1::HIS3, ORC5- FRB::KanMX6, dmc1Δ::ARG4*

yGV2397 *yGV864, [pGAD], [pGBD-PCH2-2-60]*

yGV2401 *yGV864, [pGAD], [pGBDU-PCH2-2-27]*

yGV2402 *yGV864, [pGAD-ORC1], [pGBDU-PCH2-2-27]*

yGV2741  *MATa, ho::LYS2, lys2, leu2::hisG, his4X::LEU2-URA3, his3::hisG, ura3, trp1: 3xFlag-6XGLY-Pch2::TRP1, pch2Δ::KanMX, ARG4*

*MATalpha, ho::LYS2, lys2, leu2::hisG, his4X::LEU2-URA3, his3::hisG, ura3, trp1: 3xFlag-6XGLY-Pch2::TRP1, pch2Δ::KanMX, ARG4*

yGV2760 *MATalpha, ho::LYS2, lys2, leu2::hisG, his4X::LEU2-URA3, his3::hisG, ura3, dmc1Δ::ARG4, trp1:pPCH2-3xFlag-6XGLY-deltaNTD- Pch2::TRP1, pch2Δ::KanMX, ARG4*

*MATa, ho::LYS2, lys2, leu2::hisG, his4X::LEU2-URA3, his3::hisG, ura3, dmc1Δ::ARG4, trp1:pPCH2-3xFlag-6XGLY deltaNTD-Pch2::TRP1, pch2Δ::KanMX, ARG4*

yGV2813 *MATa, ho::LYS2, lys2, leu2::hisG, his4XΔ::LEU2-URA3, his3::hisG, ura3, trp1: 3xFlag-6XGLY-Pch2-243-564::TRP1, pch2Δ::KanMX, ARG4, orc1::ORC1-TAP::HIS3*

*MATalpha, ho::LYS2, lys2, leu2::hisG, his4X ::LEU2-URA3, his3::hisG, ura3, trp1: 3xFlag-6XGLY-Pch2-243-564::TRP1, pch2Δ::KanMX, ARG4, orc1::ORC1-TAP::HIS3*

yGV2816  *MATa, ho::LYS2, lys2, leu2::hisG, his4X::LEU2-URA3, his3::hisG, ura3, trp1: 3xFlag-6XGLY -Pch2::TRP1, pch2Δ::KanMX, ARG4, orc1::ORC1- TAP::HIS3*

*MATalpha, ho::LYS2, lys2, leu2::hisG, his4X::LEU2-URA3, his3::hisG, ura3, trp1: 3xFlag-6XGLY-Pch2::TRP1, pch2Δ::KanMX, ARG4, orc1::ORC1-TAP::HIS3*

yGV2878 *MATa, ho::LYS2, lys2, leu2::hisG, his4X::LEU2-URA3, his3::hisG, ura3, trp1::hisG,  pch2Δ::KanMX, ARG4, trp1:pPch2-3xFlag-6XGLY-Pch2 E399Q::TRP1*

*MATalpha, ho::LYS2, lys2, leu2::hisG, his4X::LEU2-URA3, his3::hisG, ura3, trp1::hisG,  pch2Δ::KanMX, ARG4, trp1:pPch2-3xFlag-6XGLY-Pch2 E399Q::TRP1*

yGV2973  *MATa, ho::LYS2, lys2, leu2::hisG, his3::hisG, ura3, trp1::hisG, pch2Δ::KanMX, trp1:pPCH2-3xFlag-6XGLY FL Pch2::TRP1, his4X::LEU2- URA3, ura3, arg4-nsp, dmc1Δ::ARG4*

*MATalpha, ho::LYS2, lys2, leu2::hisG, his3::hisG, ura3, trp1::hisG, his4B::LEU2, pch2Δ::KanMX, trp1:pPCH2-3xFlag-6XGLY-Pch2::TRP1, dmc1Δ::ARG4*

*yGV3300 MATalpha, ho::LYS2, lys2, ura3, leu2::hisG, his3::hisG, trp1::hisG, RPL13A- 2XFKBP12::TRP1, fpr1::KanMX4, tor1-1::HIS3, ORC5-FRB::KanMX6, pch2::URA3:pPCH2(300bp):3HA-PCH2*

*MATa, ho::LYS2, lys2, ura3, leu2::hisG, his3::hisG, trp1::hisG, RPL13A- 2XFKBP12::TRP1, fpr1::KanMX4, tor1-1::HIS3, ORC5-FRB::KanMX6, pch2::URA3:pPCH2(300bp):3HA-PCH2*

yGV3324 *MATa, ho::LYS2, lys2, ura3, leu2::hisG, his3::hisG, ARG4, trp1::hisG, RPL13A-2XFKBP12::TRP1, fpr1::KanMX4, tor1-1::HIS3, ORC2- FRB::KanMX6, pch2::URA3:pPCH2(300bp):3HA-PCH2*

*MATalpha, ho::LYS2, lys2, ura3, leu2::hisG, his3::hisG, ARG4 , trp1::hisG, RPL13A-2XFKBP12::TRP1, fpr1::KanMX4, tor1-1::HIS3, ORC2- FRB::KanMX6, pch2::URA3:pPCH2(300bp):3HA-PCH2*

yGV3338  *MATa, ho::LYS2, lys2, ura3, leu2::hisG, TRP, HIS3, ARG4, pch2::URA3:pPCH2(300bp):3HA-pch2-E399Q, cdc6::KanMX6::pSCC1:CDC6*

MATalpha, ho::LYS2, lys2, ura3, leu2::hisG, TRP, HIS3/his3::hisG,

his4B::LEU2, ARG4, pch2::URA3:pPCH2(300bp):3HA-pch2-E399Q, cdc6::KanMX6::pSCC1:CDC6

yGV3358 *MATa, ade2-1, ura3-1, his3-11,15, trp1-1, leu2-3,112, can1-100, bar1::HYG, pep4::KanMX, TRP1::gal1-10-ORC5, ORC6, HIS3::gal1-10-ORC3, ORC4, URA3::gal1-10-CBP-TEV-ORC1, ORC2*

yGV3415 *MATa, ho::LYS2, lys2, ura3, leu2::hisG, TRP, HIS3, arg4-Bgl II,*

pch2::URA3:pPCH2(300bp):3HA-pch2-E399Q, ORC5-TAP::HIS3,  orc1::orc1-161

MATalpha, ho::LYS2, lys2, ura3, leu2::hisG, TRP, HIS3, ARG4,

pch2::URA3:pPCH2(300bp):3HA-PCH2-E399Q, ORC5-TAP::HIS3,  orc1::orc1-161

yGV3778 *yGV864, [pGAD], [pGBD-PCH2-2-91]*

yGV3779 *yGV864, [pGAD], [pGBD-PCH2-2-121]*

yGV3780 *yGV864, [pGAD], [pGBD-PCH2-2-233]*

yGV3781 *yGV864, [pGAD], [pGBD-PCH2-2-257]*

yGV3782 *yGV864, [pGAD], [pGBD-PCH2-2-270]*

yGV3791 *yGV864, [pGAD-ORC1], [pGBD-PCH2-2-233]*

yGV3793 *yGV864, [pGAD-ORC1], [pGBD-PCH2-2-121]*

yGV3802 *yGV864, [pGAD-ORC], [pGBD-PCH2-2-91]*

yGV3803 *yGV864, [pGAD-ORC], [pGBD-PCH2-2-60]*

yGV3823 *yGV864, [pGAD-ORC1], [pGBD-PCH2-2-270]*

yGV3824 *yGV864, [pGAD-ORC1], [pGBD-PCH2-2-257]*

yGV3920 *MATa, ho::LYS2, lys2, ura3, leu2::hisG, TRP/trp1::hisG, HIS3, his4B::LEU2, arg4-Bgl II, pch2::URA3:pPCH2(300bp):3HA-pch2-E399Q, orc1::orc1-161*

MATalpha, ho::LYS2, lys2, ura3, leu2::hisG, TRP1, HIS3, his4B::LEU2, arg4-Bgl II, pch2::URA3:pPCH2(300bp):3HA-pch2-E399Q, orc1::orc1-161

yGV3968 yGV864, [pGAD-ORC2], [pGBD]

yGV3974 yGV864, [pGAD-ORC2], [pGBD-PCH2]

yGV3976 *yGV864, [pGAD-ORC3], [pGBD-PCH2]*

yGV3977 *yGV864, [pGAD-ORC3], [pGBD]*

yGV3978 *yGV864, [pGAD-ORC4], [pGBD-PCH2]*

yGV3979 y*GV864, [pGAD-ORC4], [pGBD]*

yGV3980 *yGV864, [pGAD-ORC6], [pGBD-PCH2]*

yGV3981 *yGV864, [pGAD-ORC6], [pGBD]*

yGV4033  *MATa, ho::LYS2, lys2, leu2::hisG, his4X::LEU2-URA3, his3::hisG, ura3, trp1:pPCH2-3xFlag-6XGLY-Pch2 243-564::TRP1, pch2::KanMX, ARG4*

*MATalpha, ho::LYS2, lys2, leu2::hisG, his3::hisG, URA3, trp1:pPCH2- 3xFlag-6XGLY-Pch2 243-564::TRP1, pch2Δ::KanMX, ARG4, ndt80Δ::LEU2*

yGV4411 *MATalpha, ho::LYS2, lys2, ura3, leu2::hisG, his3::hisG, trp1::hisG,  RPL13A- 2XFKBP12::TRP1, fpr1::KanMX4, tor1-1::HIS3, ORC2-FRB::KanMX6, orc1::ORC1-TAP::HIS3*

*MATa, ho::LYS2, lys2, ura3, leu2::hisG, his3::hisG, trp1::hisG,  RPL13A- 2XFKBP12::TRP1, fpr1::KanMX4, tor1-1::HIS3, ORC2-FRB::KanMX6, orc1::ORC1-TAP*::HIS3

yGV4515 *MATa, ho::LYS2, lys2, ura3, leu2::hisG, his3::hisG, ARG4, trp1::hisG, RPL13A-2XFKBP12::TRP1, fpr1::KanMX4, tor1-1::HIS3, ORC2- FRB::KanMX6, pch2::URA3:pPCH2(300bp):3HA-PCH2, orc1::ORC1- TAP::HIS3*

*MATalpha, ho::LYS2, lys2, ura3, leu2::hisG, his3::hisG, ARG4 , trp1::hisG, RPL13A-2XFKBP12::TRP1, fpr1::KanMX4, tor1-1::HIS3, ORC2- FRB::KanMX6, pch2::URA3:pPCH2(300bp):3HA-PCH2, orc1::ORC1-TAP::HIS3*

Yeast strains used per figure:

1B: yGV933, yGV1506 and yGV2085

1C: yGV933, yGV1506 and yGV1966

1E: yGV2086, yGV2085, yGV2155 and yGV2156

1F: yGV2878

3B: yGV2030, yGV2114, yGV2036, yGV2117, yGV2061 and yGV2116

3D: yGV2741, yGV2813, yGV2816 and yGV4033

3F: yGV2760, yGV2973

3G: yGV48, yGV2207, yGV2760 and yGV2973

5A: yGV2030, yGV2114, yGV3782, yGV3823, yGV3781, yGV3824, yGV2060, yGV2115, yGV3780, yGV3791, yGV2321, yGV2315, yGV2322, yGV2316, yGV3779, yGV3793, yGV3778, yGV3802, yGV2397, yGV3803, yGV2401 and yGV2402

6B: yGV2367, yGV2366 and yGV2393

6C: yGV2367, yGV2366 and yGV2393

6D: yGV48, yGV1269, yGV2367, yGV2366 and yGV2393

6E: yGV2030, yGV2114, yGV2036, yGV2117, yGV3968, yGV3974, yGV3976, yGV3977, yGV3978, yGV3979, yGV3980 and yGV3981

6G: yGV933, yGV1185, yG1508 and yGV1945

6H: yGV3324, yGV4515

S1A: yGV1506, yGV1508 and yGV1537

S1B and C: yGV2086 and yGV2085

S6A: yGV2086 and yGV3338

S6B: yGV2086 and yGV3338

S6C: yGV48, yGV1269 yGV2345 and yGV1192

S8A: yGV2086, yGV3920, yGV2156 and yGV3415

S8B: yGV3324, yGV4515

S9A: yGV2203, yGV4411

S9B: yGV3324, yGV4515

S9C-F: yGV3300, yGV3324
